# Proceedings: Predictive value of acid phosphatase.

**DOI:** 10.1038/bjc.1974.179

**Published:** 1974-08

**Authors:** B. Morgan


**PREDICTIVE VALUE OF ACID PHOSPHATASE.** B. MORGAN. Department of Chemical Pathology, University of Leeds.

There is increasing interest in the use of clinical and biochemical data to assess the probability that a patient has, or will develop, a particular state. The complexity of these techniques arises in part because they are based on many variables measured in each individual. Yet the aims of these techniques are identical with the aims of making a single measurement in each individual. It is these aims which will be discussed here in relation to a single variable, namely the serum acid phosphatase activity, and a single clinical condition, namely carcinoma of the prostate.

The *ideal situation* would have been as follows: (1) Two groups were defined which were comparable in all ways except that one group had carcinoma of the prostate gland (CA) and the other did not (N); (2) There was a measurement (*acid phosphatase*) which was simpler than the techniques used to separate the initial groups and which had different values in the groups CA and N; (3) There was no overlap in the values of X in the groups CA and N; (4) There was no other condition which could be confused with carcinoma of the prostate.

The real life situation is of course far from this ideal. One fundamental difference is that there is no complete separation of the values of serum acid phosphatase (AP) in the two groups (N and CA). Various changes in technique have been suggested in order to diminish this overlap of AP between the groups. These changes are largely aimed at improving organ specificity by the choice of substrate and the addition of isoenzyme inhibitors (Bodansky, *Clin. Chem.* 1972, **15**, 43; Schwartz, *Clin. Chem.*, 1973, **19**, 10).

While these attempts will no doubt continue, it seems reasonable to conclude that in this clinical situation, as in so many others, a single measurement will not completely discriminate between the groups and that overlap of the results will remain.

What is so commonly done in practice is to define a value (the upper limit of normal) to assume that values above this will not occur in healthy persons. The finding of a high value then " indicates " the presence of the disorder. This approach is an attempt to make the situation like the ideal one with complete discrimination.

However, this approach makes no use of the information in the absolute value of the measurement. Thus, the higher the value of AP, the greater the probability of carcinoma of the prostate. This probability is rarely formally defined for a single variable although it is now part of some complex statistical analyses involving several variables (Hartz, *Clin. Chem.*, 1973, **19**, 113). This probability function must also take into account the relative prevalence (probabilities) of the disorders or states which are being discriminated.


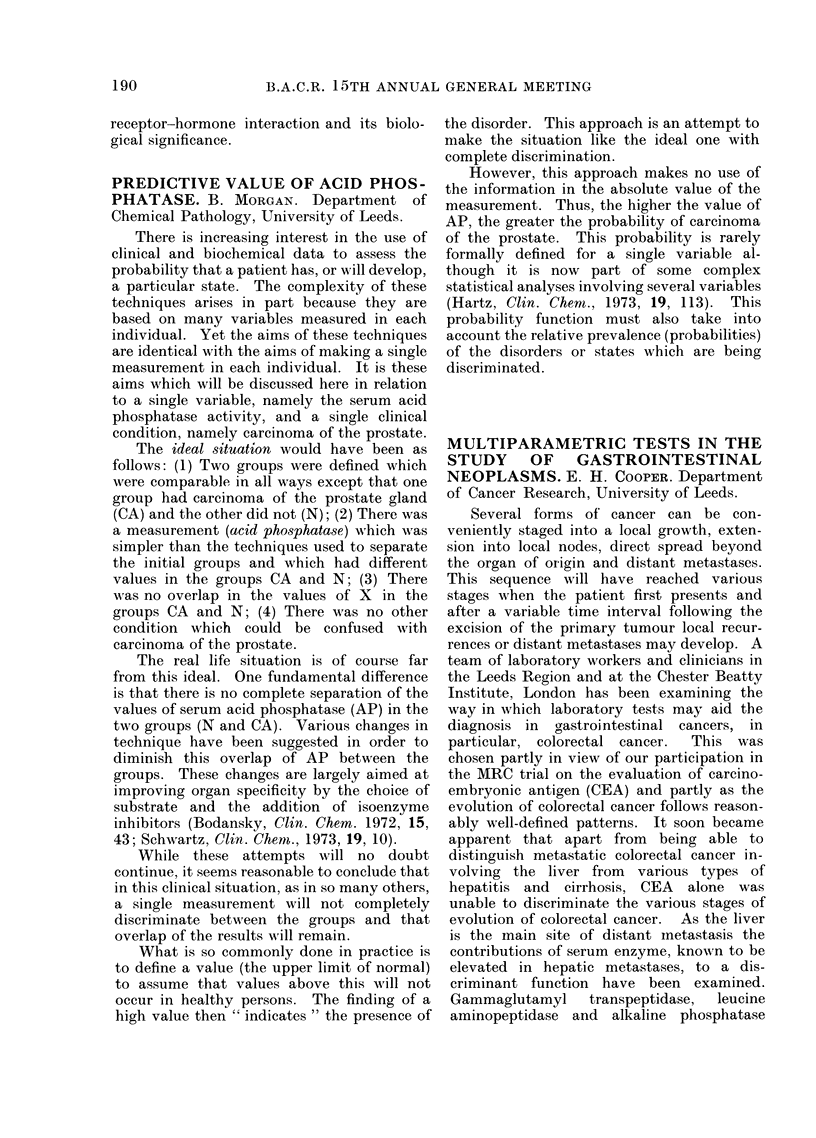


## References

[OCR_00072] Hartz S. C. (1973). A statistical model for assessing the need for medical care in a health screening program.. Clin Chem.

